# An Extremely Rare Case of Advanced Metastatic Small Cell Neuroendocrine Carcinoma of Sinonasal Tract

**DOI:** 10.1155/2016/1496916

**Published:** 2016-07-27

**Authors:** Yu Yu Thar, Poras Patel, Tiangui Huang, Elizabeth Guevara

**Affiliations:** ^1^Department of Medicine, Division of Hematology and Oncology, The Brooklyn Hospital Center, Brooklyn, NY 11201, USA; ^2^Department of Medicine, The Brooklyn Hospital Center, Brooklyn, NY 11201, USA; ^3^Department of Pathology, The Brooklyn Hospital Center, Brooklyn, NY 11201, USA

## Abstract

Small cell neuroendocrine carcinoma (SNEC) is a rare form of malignancy. It mainly presents as bronchogenic neoplasm, and the extrapulmonary form accounts for only 0.1% to 0.4% of all cancers. These extrapulmonary tumors have been described most frequently in the urinary bladder, prostate, esophagus, stomach, colon and rectum, gall bladder, head and neck, cervix, and skin. Primary SNEC of the sinonasal tract is extremely rare with only less than 100 cases reported in the literature. Because of extreme rarity and aggressiveness of the tumor, the management for this entity varies considerably mandating multimodality approach. In this paper, we report a patient presented with left-sided facial swelling, and the histopathologic examination confirmed primary SNEC of left sinonasal tract. The tumor involved multiple paranasal sinuses with invasion into the left orbit and left infratemporal fossa and metastasized to cervical lymph nodes and bone. The patient encountered devastating outcome in spite of optimal medical management and treatment with palliative chemotherapy highlighting the necessity for further research of primary SNEC of head and neck.

## 1. Introduction

Neuroendocrine tumors constitute broad spectrum of malignant epithelial neuroendocrine neoplasms. They are further subdivided into typical carcinoid (well-differentiated), atypical carcinoid (moderately differentiated), and small cell carcinoma (poorly differentiated neuroendocrine carcinoma) [1]. Small cell neuroendocrine carcinoma (SNEC) was first described in the 19th century in the context of lung cancer. Head and neck SNEC have been described only since 1965 [2]. The most common site of head and neck neuroendocrine carcinoma is the larynx. Primary neuroendocrine carcinomas of nasal and paranasal cavities are extremely rare and less than 100 cases have been in the medical literature. It is a highly proliferative epithelial neuroendocrine tumor with an aggressive behavior that is characterized by early, widespread metastases via the lymphatic as well as the blood stream [2]. Because of the rarity of this neoplasm, there are no specific recommendations pertaining to the management and treatment options are generally extrapolated from similar tumors of pulmonary origin. In this case report, we have presented a patient who was diagnosed with an extremely rare poorly differentiated (small cell) neuroendocrine carcinoma of sinonasal tract with metastasis. We have also described the literature review of clinical presentation, imaging characteristics and pathologic features, and, most importantly, the management of SNEC of head and neck.

## 2. Case Report

A 54-year-old African-American chronic smoker woman presented to Emergency Department with worsening left-sided facial swelling and blurry vision for one week. Five months ago, the patient was evaluated for left neck mass with aspiration at the other facility. The pathology was benign and she was treated for infection with antibiotics. Then, the patient failed to follow up. Since then, the patient noted intermittent swelling of left neck mass for several months which for past one week has progressively gotten worse, associated with periorbital swelling, left-sided blurry vision, and left nasal congestion and epistaxis. She had 10–15 pounds of unintentional weight loss over last couple of months with increasing fatigue. She has a known history of HIV (noncomplaint with antiretroviral therapy), asymptomatic hepatitis B virus carrier, and chronic kidney disease.

Comprehensive physical examination revealed a nontendered, nonmobile, hard, large left-sided neck mass with left-sided facial and orbital swelling. She was also noted to have dry mouth, bilateral pale conjunctiva, mass in left nasal chamber, excessive lacrimation, intermittent alternating exotropia, and restricted left eye extraocular movement. Systemic review demonstrated no organomegaly and no other lymphadenopathy.

**Figure 1:**
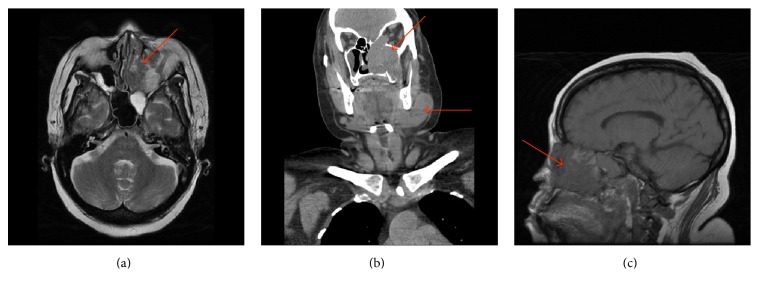
((a) (axial), (b) (coronal), and (c) (sagittal)) CT scan of the neck showing extensive lymphadenopathy, left greater than right, and extensive soft tissue involving the left nasal cavity and multiple paranasal sinuses wit invasion into the left orbit and left infratemporal fossa.

**Figure 2:**
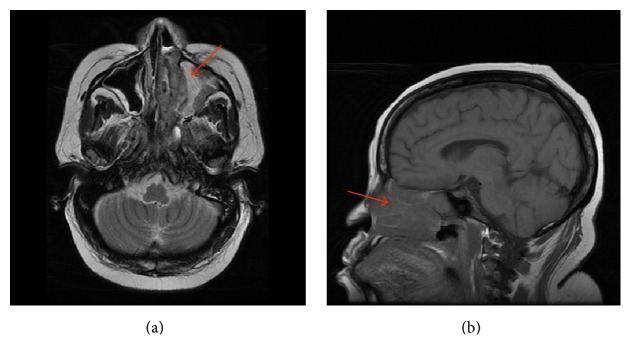
((a) (axial) and (b) (sagittal)) MRI of the brain showing a soft tissue mass in the left maxillary sinus and left nasal cavity with extension to involve the medical left orbit.

**Figure 3:**
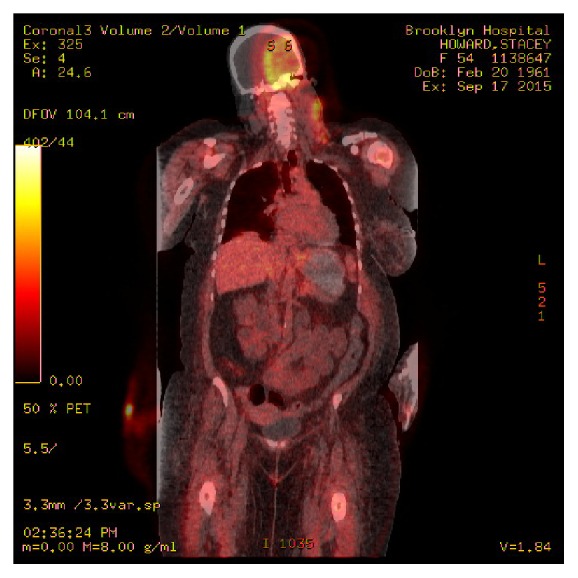
Positron Emission Tomography/Computed Tomography (PET/CT) showing minimal FDG uptake at the large mass lesion at the left ethmoid and maxillary sinuses, moderate to significant FDG uptake at bilateral cervical lymph nodes, and minimal heterogeneous FDG uptake along the thoracolumbar vertebra and pelvic bones.

**Figure 4:**
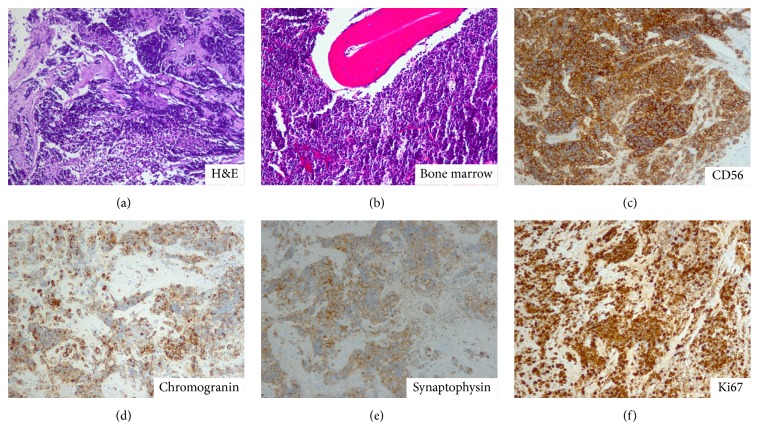
Pathology of the left nasal mass and bone marrow shows small cell undifferentiated (neuroendocrine) carcinoma. Light microscopy showed small round cells with scanty cytoplasm and hyperchromatic nuclei ((a) and (b)). Immunohistochemical staining showing tumor positivity for CD56 (c), chromogranin (d), and synaptophysin (e) (IHC ×100) and nuclear positivity to Ki-67 (f) (IHC ×100).

Laboratory results were significant for anemia, thrombocytopenia, elevated lactate dehydrogenase, and uric acid levels. Computerized tomography (CT) scan of neck showed extensive lymphadenopathies, left greater than right, and a soft tissue mass involving left nasal cavity and multiple paranasal sinuses with invasion into left orbit and left infratemporal fossa (Figures 1(a) and 1(b)). The patient proceeded incisional biopsy of the left nasal mass. Histopathology described the tumor cells were small with little cytoplasm and a high nuclear/cytoplasmic ratio, arranged in sheets with both scattered and geographic necrosis. The nuclei were oval to spindle-shaped and pleomorphic with absent or indistinct nucleoli. Mitotic figures were numerous (Ki-67 is present). Immunohistochemical profile showed tumor cells positivity for cytokeratin AE1/AE3, Cam5.2, Epithelial Membrane Antigen (EMA) and positivity for neuroendocrine markers including CD56, chromogranin, synaptophysin, and neuron-specific enolase (NSE) (Figure 4). The tumor cells were also positive for Bcl-2 but were negative for lymphoid markers such as CD3, CD5, CD7, CD10, CD79a, CD20, and CD30. They were also negative for Thyroid Transcription Factor-1 (TTF-1), Napsin A, Epstein-Barr virus (EBV), Bcl-1, CD99, S100, and vimentin. The diagnosis of a small cell neuroendocrine carcinoma was made. Bone marrow biopsy disclosed infiltration of monomorphic small blue cells diffusely positive for CD56, consistent with bone marrow involvement of nasal small cell neuroendocrine carcinoma. CT scan of the chest, abdomen, and pelvis was unremarkable. Magnetic resonance imaging (MRI) study of the brain demonstrated soft tissue mass in the left maxillary sinus and left nasal cavity with extension to involve the medial left orbit. No intracranial mass lesion was found (Figures 2(a) and 2(b)). Positron Emission Tomography/Computed Tomography (PET/CT) disclosed a large destructive mass involving the left nasal cavity, paranasal sinuses, and left orbit with extensive bilateral cervical adenopathy, all of which were hypermetabolic (Figure 3). The clinical TNM (tumor node metastasis) stage of the patient was T4N2M1 (stage IV) according to the American Joint Committee on Cancer (AJCC) [3].

During the course, the patient had acute chronic renal failure and worsening of hematological parameters requiring hemodialysis and blood products, respectively. Given the extent of her disease, the patient was started on palliative chemotherapy with carboplatin (etoposide was omitted due to thrombocytopenia) urgently as inpatient. The patient was not felt to be a candidate for cisplatin based chemotherapy due to her renal dysfunction. Treatment with combined carboplatin and etoposide was planned initially. But etoposide was omitted due to severe thrombocytopenia. There was a good clinical response after receiving the first cycle of chemotherapy; however, due to worsening anemia and thrombocytopenia, further chemotherapy was delayed. Unfortunately, the patient succumbed to the disease. The autopsy was performed and there was no definite mass found below the neck. Extensive sampling of lung, gastrointestinal tract including liver and spleen, and genitourinary tract were unremarkable. Only the vertebra showed small blue cells which was further confirmed by CD56 stain.

## 3. Discussion

Small cell undifferentiated neuroendocrine carcinomas of sinonasal tract, also known as poorly differentiated neuroendocrine carcinomas, are extremely rare and represent a histological spectrum of differentiation. It has been reported to be highly aggressive with very poor prognosis. Most prevailing malignancy of head and neck is squamous cell carcinoma, followed by adenocarcinoma [2]. Larynx is the most common site for neuroendocrine carcinoma in the head and neck, whereas paranasal sinuses accounts for approximately 0.3% of all cancers. The mean age at presentation is approximately 50 years (range: 26–77 years) and there is no predilection for race or sex reported in the literature. Distant metastases frequently occur in lungs, liver, and bone [4, 5].

Most frequent clinical features of patients with neuroendocrine carcinoma of the head and neck, which were also seen in our case, are recurrent epistaxis, nasal obstruction, nasal discharge, proptosis, paresthesia, and anosmia. Occasionally, the presenting symptoms may be exophthalmos, facial pain, and swelling [4]. These tumors may be associated with paraneoplastic syndrome that often manifests as the syndrome of inappropriate antidiuretic hormone secretion (SIADH). However, it is an uncommon presentation for SNEC of the head and neck [6, 7]. There is no distinctive computed tomography (CT) or magnetic resonance imaging (MRI) characteristics to diagnose or differentiate nonlaryngeal neuroendocrine carcinoma in the head and neck from the most common salivary gland neoplasms.

Microscopically, it is indistinguishable between SNEC of head and neck from those of bronchogenic origin. These cells are typically densely cellular, oval to spindle-shaped, and pleomorphic nuclei, frequent mitoses, and necrosis [5]. Cytokeratin (AE 1/3), chromogranin, and neuron-specific enolase, which were all positive in the immunohistochemical profile of our patient, are characteristic tumor markers of epithelial and neuroendocrine differentiation [8]. EBV RNA is negative by in situ hybridization [9]. Cytokeratin is the most useful marker to differentiate between neural and epithelial neuroendocrine tumors. The use of immunostains, electromicroscopy, and molecular genetics has helped to understand this lesion, but the mainstay of diagnosis of this tumor remains to be the light microscopy [4, 10]. SNEC is exceptionally rare and it is important to differentiate from sinonasal undifferentiated carcinoma (SNUC) which are distinctly larger, with more prominent eosinophilic cytoplasm and larger nuclei, often with prominent nucleoli by light miscopy [11]. Immunohistochemistry is very useful in distinguishing small cell neuroendocrine carcinoma from basaloid squamous cell carcinoma and adenocystic carcinoma, which can be difficult to distinguish on small biopsy specimen. It is very important for management and prognosis to differentiate small cell neuroendocrine carcinoma from the more common nasopharyngeal squamous cell carcinoma [12, 13].

As witnessed in our case, tumors of this type are often disseminated at diagnosis; thus, it is essential to perform thorough metastatic workup before initiating the treatment. Multimodality therapy is increasingly used in small cell neuroendocrine carcinoma as it is an aggressive malignancy with high rates of local recurrence and metastases. Treatment modalities include chemotherapy, radiotherapy, and possibly surgery, depending on the extent of disease or the primary site. Surgical option is reserved for local relapse with no evidence of metastases because surgical results for this tumor have been disappointing. The combination of chemotherapy and radiation therapy, with or without surgery, has been recommended since late 1990s [9, 14]. Platinum-based chemotherapy followed by radiotherapy is a well known recommendation that has been tested in a large study. Due to high rates of intracranial metastases in patients with small cell neuroendocrine cancer of the nasal and paranasal sinuses, studies have suggested that it should be treated with systemic chemotherapy and radiotherapy and prophylactic cranial irradiation [7]. Prognosis remains poor despite combined modality treatment. More extensive studies are needed to assess the optimal management and development of standardized treatment protocols.

## 4. Conclusion

SNEC of sinonasal tract is an uncommon neoplasm with tendency toward recurrence and distant metastasis. Because of its rarity and aggressive nature, diagnosis and management of SNEC remain a challenge. Further research is needed to develop more specific, targeted approach of SNEC of head and neck in order to improve patient's survival and quality of life.
